# PP14 Disparities In Quality-Adjusted Life Expectancy Associated With Educational Attainment: Population Norms For Australia

**DOI:** 10.1017/S0266462325102341

**Published:** 2025-12-29

**Authors:** Sheridan Rodda, Melanie Lloyd, Jedidiah Morton, Jennifer Welsh, Rosemary Korda, Zanfina Ademi

## Abstract

**Introduction:**

Summary measures of health, such as quality-adjusted life expectancy (QALE), are used to monitor health inequalities by reflecting both quantity and quality of life. The Australian distribution of QALE by socioeconomic status, measured using educational attainment, is unknown. We aimed to estimate QALE stratified by sex and year of age across levels of educational attainment for the Australian population.

**Methods:**

Educational attainment was measured as: low (secondary schooling incomplete), intermediate (completed secondary schooling and/or non-tertiary qualification), or high (completed bachelor’s degree or above). The Person-Level Integrated Data Asset linked Australian Census (2016) demographic information to Deaths Registrations (2016 to 2019). Age-sex-education specific mortality rate ratios were then applied to the 2019 population. Life tables were constructed to generate life expectancy (LE). The Household, Income and Labour Dynamics in Australia Survey (2022) was used to estimate mean Short Form 6 Dimensions (SF-6D) health utility by age, sex, and education using linear regression. Person-years lived were multiplied by utility to calculate QALE.

**Results:**

Both LE and QALE increased with increasing level of education. Males aged 25 years with a high level of education had a LE of 61.0 years and a QALE of 39.9 (95% confidence interval [CI]: 38.7, 41.2) years, compared with 53.7 years and 28.8 (95% CI: 27.4, 30.3) years, respectively, for those with low education, a difference of 11.1 quality-adjusted life years (39%). Females with high educational attainment had a LE of 63.1 years and a QALE of 36.9 (95% CI: 35.8, 38.1) years, compared with 59.2 years and 29.3 (95% CI: 27.9, 30.8) years, respectively, for low education, a QALE difference of 7.6 years (26%).

**Conclusions:**

There were large disparities in QALE, impacting both the quantity and quality of life, associated with educational achievement in Australia. Tailored policies addressing education-related inequalities in health are needed. The QALE distribution across equity relevant groups provided in this study can be utilized in future equity informative studies that evaluate the equity impact of healthcare funding decisions.

